# Creutzfeldt-Jakob Disease variant presenting with prominent basal ganglia imaging

**DOI:** 10.31744/einstein_journal/2025AI0807

**Published:** 2025-02-07

**Authors:** Denison Alves Pedrosa, Rafael Bernhart Carra, Natália Merten Athayde, Karina Silveira Massruha, Rachel Marin de Carvalho, René de Araújo Gleizer

**Affiliations:** 1 Hospital Israelita Albert Einstein São Paulo SP Brazil Hospital Israelita Albert Einstein, São Paulo, SP, Brazil.; 2 Universidade de São Paulo São Paulo SP Brazil Universidade de São Paulo, São Paulo, SP, Brazil.; 3 Hospital Israelita Albert Einstein Hospital Municipal Dr. Moysés Deutsch São Paulo SP Brazil Hospital Municipal Dr. Moysés Deutsch; Hospital Israelita Albert Einstein, São Paulo, SP, Brazil.

**Figure 1:**
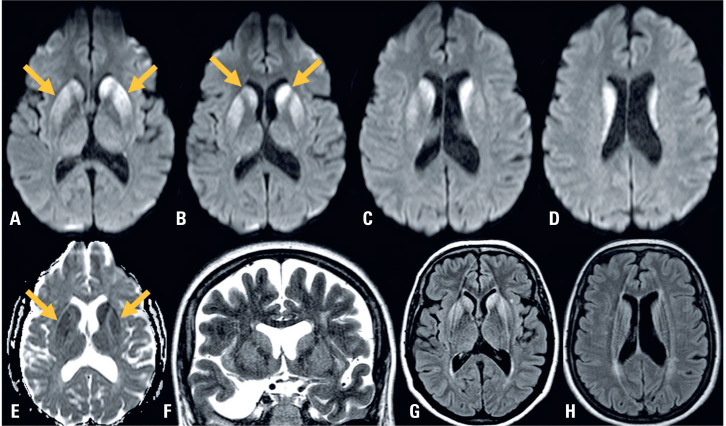
Axial diffusion-weighted imaging (A–D) showing hyperintensity involving the bilateral putamen (yellow arrows) and caudate heads (yellow arrows), with hypointensity on the apparent diffusion coefficient map (yellow arrows in E), hyperintensity on coronal T2-weighted imaging (F), and axial T2 fluid-attenuated inversion recovery (FLAIR, G, H). No cortical changes were observed on any of the MRI sequences

A 53-years old white women was presented with a four-weeks history of unsteady gait, followed by mental confusion and progressive memory loss. Neurological examination revealed axial ataxia, short-term memory loss, and brisk deep tendon reflexes in all limbs. Brain MRI (Figure 1) revealed a diffusion signal abnormality involving the bilateral caudate heads and putamen, with no cortical changes. Cerebrospinal fuid (CSF) analysis showed 1 cells per μL, protein content of 25.5 mg/dL, and glucose content of 58 mg/dL. Eletroencefalograma revealed no periodic sharp wave complexes. Real-time quaking-induced conversion (RT-QuIC) test and 14-3-3 protein detection in the CSF were both positive. Eight-weeks later, multifocal myoclonus developed, and death occurred shortly thereafter.

Sporadic Creutzfeldt-Jakob disease (CDJ) is a fatal, rapidly progressive neurodegenerative disease that was first described as a dementia syndrome associated with cortical, striatal, and spinal cord involvement.^(1)^ Its pathogenesis is related to the alteration of a naturally existing prion protein (PrPc) to an abnormal folder protein termed scrapie prion protein (PrPSc), and its clinical presentation can vary.^(2)^ Brain MRI findings, especially those derived from diffusion-weighted imaging, play a pivotal role in recognizing and distinguishing sCJD from alternative diagnoses. Abnormal cortical signal intensity on MRI exhibits sensitivity, specificity, and accuracy exceeding 90% for sCJD.^(3)^ However, abnormalities in the deep gray matter represent atypical MRI findings are even rarer.^(4)^ Our case was characterized by significant basal ganglia imaging and less cortical involvement, which may pose challenges in cases with similar presentations.
